# Synthesis and Characterization of Poly(Ethylene Glycol) Based Thermo-Responsive Hydrogels for Cell Sheet Engineering

**DOI:** 10.3390/ma9100854

**Published:** 2016-10-20

**Authors:** Kuk Hui Son, Jin Woo Lee

**Affiliations:** 1 Department of Thoracic and Cardiovascular Surgery, Gachon University Gil Medical Center, Gachon University, 34, Namdong-daero 774beon-gil, Namdong-gu, Incheon 21565, Korea; dr632@gilhospital.com; 2 Department of Molecular Medicine, School of Medicine, Gachon University, 155 Gaetbeol-ro, Yeonsu-ku, Incheon 21999, Korea

**Keywords:** *N*-isopropylacrylamide (NIPAm), poly(ethylene glycol) diacrylate (PEGDA), thermo-responsive hydrogel, cell sheet, tissue engineering

## Abstract

The swelling properties and thermal transition of hydrogels can be tailored by changing the hydrophilic-hydrophobic balance of polymer networks. Especially, poly(*N*-isopropylacrylamide) (PNIPAm) has received attention as thermo-responsive hydrogels for tissue engineering because its hydrophobicity and swelling property are transited around body temperature (32 °C). In this study, we investigated the potential of poly(ethylene glycol) diacrylate (PEGDA) as a hydrophilic co-monomer and crosslinker of PNIPAm to enhance biological properties of PNIPAm hydrogels. The swelling ratios, lower critical solution temperature (LCST), and internal pore structure of the synthesized p(NIPAm-*co*-PEGDA) hydrogels could be varied with changes in the molecular weight of PEGDA and the co-monomer ratios (NIPAm to PEGDA). We found that increasing the molecular weight of PEGDA showed an increase of pore sizes and swelling ratios of the hydrogels. In contrast, increasing the weight ratio of PEGDA under the same molecular weight condition increased the crosslinking density and decreased the swelling ratios of the hydrogels. Further, to evaluate the potential of these hydrogels as cell sheets, we seeded bovine chondrocytes on the p(NIPAm-*co*-PEGDA) hydrogels and observed the proliferation of the seed cells and their detachment as a cell sheet upon a decrease in temperature. Based on our results, we confirmed that p(NIPAm-*co*-PEGDA) hydrogels could be utilized as cell sheets with enhanced cell proliferation performance.

## 1. Introduction

Hydrogels are three-dimensionally cross-linked networks of polymers that can imbibe large quantities of aqueous solutions. Some of them have also shown abilities to respond to various external stimuli such as temperature, pH, solvent, ionic strength, and electric field, or to combinations of these stimuli [1,2,3,4,5].

Poly(*N*-isopropylacrylamide) (PNIPAm) is one of the most investigated thermo-responsive hydrogels and exhibits a lower critical solution temperature (LCST) of around 32 °C in an aqueous solution. Below the LCST, the polymer is soluble and imbibes water molecules owing to hydrophilic interactions. However, at temperatures above the LCST, the polymer undergoes a phase separation and expels water molecules out from the polymer networks due to the dominant hydrophobic interactions between isopropyl groups [6,7]. Until now, many studies have been conducted to manipulate the LCST of PNIPAm to temperatures close to the physiological temperature by copolymerizing it with hydrophilic or hydrophobic co-monomers [4,8,9,10,11,12,13].

Poly(ethylene glycol) (PEG)-based hydrogels have been extensively explored as three-dimensional tissue engineering scaffolds. They have also found other biomedical applications and are used in drug-controlled release matrices due to their excellent biocompatibility, hydrophilicity, and ability to prevent protein adsorption and cell adhesion [14,15]. Zhang et al. synthesized p(NIPAm-*co*-PEGDA) microsphere hydrogels as a potential drug release matrix and demonstrated that the swelling ratio and particle size were dependent on the solvent composition [16]. Lee and co-workers also copolymerized NIPAm with *N*,*N*’-methylenebisacrylamide (MBAm), ethylene glycol dimethacrylate (EGDMA), and tetra(ethylene glycol) dimethacrylate (TEGDA) and found that the highest swelling ratios were observed with TEGDA as it had the highest hydrophilicity among the various crosslinkers employed [17,18]. Previously, Okano and his coworkers have studied the potential application of such hydrogels as thermo-responsive monolayer cell culture substrates by taking advantage of the thermo-responsive characteristics of PNIPAm [19]. The authors found that a PNIPAm-grafted surface can easily be altered by changing the surrounding temperature and that the surface can provide a new way of harvesting intact cell sheets without using any enzymatic agents [20,21,22].

In this study, we synthesized NIPAm-based thermo-responsive hydrogels via copolymerization using PEGDA as a co-monomer, which can have a dual function as a hydrophilic moiety as well as a crosslinker. We then systematically investigated the effect of the hydrophilic-hydrophobic balance, which was manipulated by changing the weight ratios between NIPAm and PEGDA and by varying the molecular weight of PEGDA, on the swelling behaviors of the p(NIPAm-*co*-PEGDA) hydrogels. In addition, we evaluated the potential of the p(NIPAm*-co-*PEGDA) hydrogels for use as 2D monolayer cell culture substrates and tested their ability to provide structural support for the seeded chondrocytes and to detach the proliferated cells as an intact cell sheet upon a decrease in the surrounding temperature.

## 2. Experimental

### 2.1. Materials

NIPAm (*N*-isopropylacrylamide) was purchased from TCI America (Portland, OR, USA). Toluene and triethylamine were purchased from Fisher Scientific (Pittsburgh, PA, USA), and dichloromethane and diethyl ether were purchased from Sigma-Aldrich (St. Louis, MO, USA). Poly(ethylene glycol) diacrylate (PEGDA) (Mn = 508) was purchased from Polyscience, Inc. (Warminster, PA, USA). PEG (Mn = 3400, 6000, 10,000 and 20,000) and acryloyl chloride were purchased from Sigma-Aldrich and were used without any further purification. Photoinitiator (Irgacure 2959) was obtained from Ciba Specialty Chemicals (Basel, Switzerland) and was dissolved in 70% ethanol to make a 10% w/v stock solution. 

### 2.2. Synthesis of PEGDA with Different Molecular Weights

Poly(ethylene glycol) diacrylate (PEGDA) oligomers of varying molecular weights (Mn = 3400, 6000, 10,000, and 20,000) were synthesized as described in [23,24]. Briefly, 18 g (3 mmol) of PEG (Mn = 6000) was dissolved in 300 mL of toluene in a 500 mL round bottom flask in an oil bath heated at 150 °C. The solution was then refluxed for 4 h under vigorous stirring. Traces of water in the reaction mixture were removed by azeotropic distillation. Upon cooling the solution to room temperature, 1.214 g (12 mmol) of triethylamine was added to it under vigorous stirring. The flask was then moved to an ice bath and stirred for 30 min. Next, 1.086 g (12 mmol) of acryloyl chloride in 15 mL of anhydrous dichloromethane was added dropwise to the reaction mixture for 30 min. Thus, the molar ratio of PEG, acryloyl chloride and triethylamine was 1:4:4. After keeping the reaction mixture in an ice bath for another 30 min, the flask was heated to 45 °C overnight. The reaction mixture was then cooled to room temperature, and the quaternary ammonium salt formed was removed from the reaction mixture by filtering through Celite^®^ 545 filter aid (2–3 cm thick) on a fritted glass funnel. The filtrate was concentrated using a rotary evaporator and then precipitated in excessive diethyl ether. The white precipitate was collected by filtration and vacuum dried at 40 °C for 24 h. The resultant PEGDA oligomer was purified by precipitation, followed by column chromatography and dialysis prior to its usage. The PEGDA (Mn = 3400, Mn = 10,000 and Mn = 20,000) were synthesized by using the same procedure. 

### 2.3. Synthesis of the p(NIPAm-co-PEGDA) Hydrogels

The hydrogels were synthesized using photopolymerization techniques. Briefly, a solution of 11.32% w/v of NIPAm and PEGDA of different molecular weights in the desired weight ratios (1:0.25 and 1:0.5) were prepared in deionized (DI) water. The nomenclature used for the various hydrogels discussed in this study is tabulated in Table 1. Then, Irgacure 2959, a photoinitiator of 0.1% w/v was added to the prepared solution. The polymerization mixture was irradiated for 5 min under 365 nm UV light at an intensity of 4.5 mW/cm^2^ (Glowmark Systems, Upper Saddle River, NJ, USA) using the mold with dimensions of 5.5 mm diameter and 3 mm height. The synthesized hydrogels were then immersed in DI water for 24 h to remove the unreacted monomers, and DI water was replaced with fresh DI water three times during the washing.

### 2.4. Nuclear Magnetic Resonance (NMR) Spectroscopic Analysis

**Figure 1:**
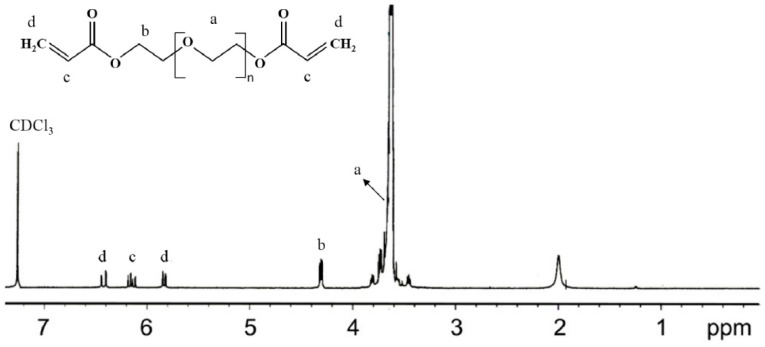
^1^H NMR spectrum of poly(ethylene glycol) diacrylate (PEGDA) (Mn = 6000). (δ, ppm): 3.6 (–OCH_2_–CH_2_–), 4.3 (–CH_2_OCO–), 5.8–6.5 (CH_2_=CH–).

The ^1^H NMR was used to verify the degree of acrylation of synthesized PEGDA (Figure 1) and all PEGDA samples used in this study have above 90% of degree of acrylation. The ^1^H NMR and ^13^C NMR spectra of the swollen p(NIPAm-*co*-PEGDA) network in deuterated water (D_2_O) were recorded on a Varian Mercury 400-MHz spectrometer using D_2_O as the solvent. Briefly, the hydrogels were synthesized and washed in DI water and then freeze-dried. The dried gels were then allowed to swell in D_2_O and crushed into a fine powder for NMR analysis.

### 2.5. Microstructure Analysis

The internal microstructure of the p(NIPAm*-co-*PEGDA) hydrogels was examined by scanning electron microscopy (SEM, JEOL, JSM-7500F, Toyko, Japan). Briefly, the samples were immersed in DI water for 24 h to allow them to reach equilibrium and were frozen rapidly in liquid nitrogen. The frozen hydrogels were then freeze-dried for 24 h using a lyophilizer, followed by gold coating using a sputter coater (Emitech K575X Sputter Coater, Quorum Technologies, Laughton, UK) for 30 s prior to SEM imaging.

### 2.6. Swelling Ratio Measurement

The equilibrium swelling ratios of the swollen hydrogels were measured using a gravimetric method. The hydrogels were washed in excess water to remove the unreacted monomers and placed in a hot air sterilizer oven (Fisher Scientific 525D, Champaign, IL, USA) at 60 °C and then in a vacuum oven (OV-11, JEIO Tech, Seoul, Korea) until a constant dried weight was obtained. Each sample was immersed in DI water at 5–50 °C for 24 h, and their swollen weights were measured immediately after the removal of excess water from the surface by blotting with slightly wet tissue paper. The equilibrium swelling ratios of the samples were then determined as a ratio of the weight of the swollen gels with an equilibrium state to that of the dried gels.

### 2.7. Kinetics of Deswelling and Reswelling

The kinetics of deswelling and reswelling were investigated by measuring the wet weights of the hydrogels as a function of time. For determining deswelling kinetics, we used the same method described earlier. Briefly, we equilibrated the hydrogels at 10 °C, transferred them to 50 °C, and measured their wet weights at different time points. 

### 2.8. LCST Measurements

The LCSTs of each of the p(NIPAm*-co-*PEGDA) hydrogels were determined as the inflection point of the plot of swelling ratios at various temperatures [25].

### 2.9. Application of the Prepared Hydrogels as 2D Monolayer Cell Culture Substrates for Cell Sheet Engineering

The potential application of the p(NIPAm*-co-*PEGDA) hydrogels for cell sheet engineering was evaluated using bovine chondrocytes (bCCs). Prior to seeding the cells, the hydrogels were sterilized with 70% ethanol and washed with fresh Phosphate Buffered Saline (PBS). The cells were then seeded onto the hydrogel sheets (equilibrated at 37 °C) at a seeding density of 7 × 10^3^ cells/cm^2^. The cell-seeded hydrogels, with a diameter of 1.6 cm and a thickness of 1 mm, were cultured in 2 mL of chondrocyte growth medium (DMEM supplemented with 10% fetal bovine serum (FBS), 0.04 mM·L^−1^ proline (Sigma-Aldrich), 50 µg/mL ascorbic acid (Sigma-Aldrich), 0.1 mM non-essential amino acid (GIBCO), 100 U/mL penicillin and 100 µg/mL streptomycin). All the cell cultures were performed at 37 °C under 5% CO_2_.

### 2.10. Characterization of the Cell Sheet

When the cells became confluent on the hydrogels, we exposed them to room temperature to see whether the intact cell sheet can be detached spontaneously upon changing the surrounding temperature. After waiting 5 min at room temperature, the detachment of the hydrogel was observed using an inverted microscope.

### 2.11. Statistical Analysis

All experiments are carried out three-times repetition (*n* = 3) and the data are presented as mean ± standard deviation (SD). Single factor analysis of variance (ANOVA) with Turkey’s Multiple Comparison Test was performed to determine the statistical significance; *p* < 0.05 was considered as statistically significant.

## 3. Results and Discussion

### 3.1. Synthesis of the p(NIPAm-co-PEGDA) Hydrogels

**Figure 2:**
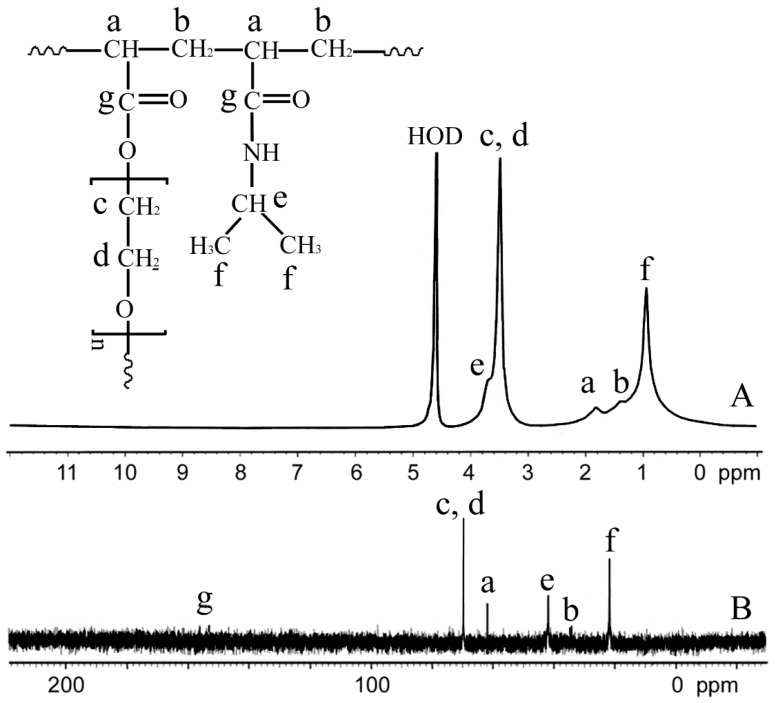
(**A**) ^1^H NMR and (**B**) ^13^C NMR spectra of the 1:0.5PEG(6000) p(NIPAm*-co-*PEGDA) networks in D_2_O. ^1^H NMR (δ, ppm): 0.97 (–CH_3_), 1.38 (–CH_2_–CH–), 1.82 (–CH_2_–CH–), 3.7 (–CH(CH_3_)_2_), 3.51 (–CH_2_–CH_2_–O–). ^13^C NMR (δ, ppm): 21.9 (–CH_3_), 34.0 (–CH_2_–CH–), 42.0 (–CH(CH_3_)_2_), 62.0 (–CH_2_–CH–), 69.8 (–CH_2_–CH_2_–O–), 152–156 (C=O).

The chemical structure of the synthesized 1:0.5PEG(6000) hydrogel was verified using ^1^H NMR and ^13^C NMR. As shown in Figure 2, the NMR spectra of the 1:0.5PEG(6000) hydrogel, synthesized by copolymerizing NIPAm and PEGDA (Mn = 6000) (at a weight ratio of 1:0.5), showed that the two co-monomers were successfully incorporated into the network.

### 3.2. Microstructure Analysis

**Figure 3:**
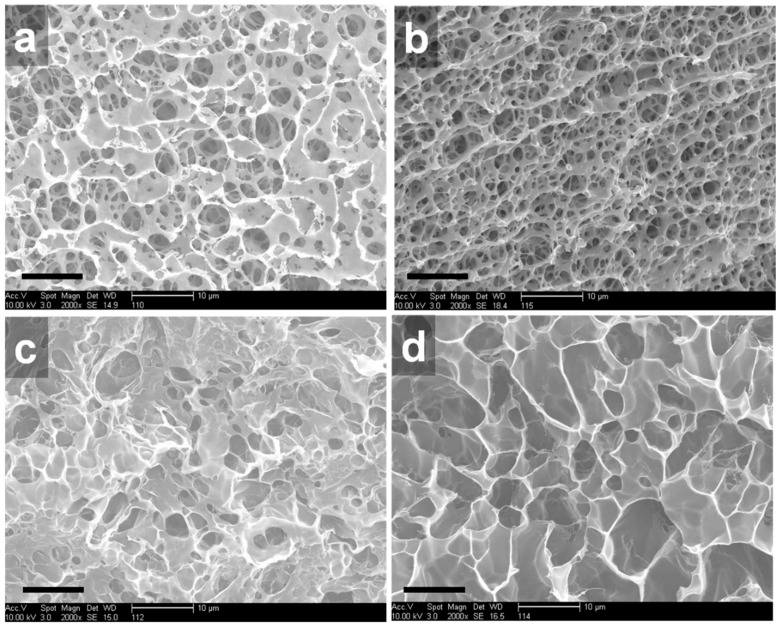
SEM images of the internal fracture surface of the p(NIPAm*-co-*PEGDA) hydrogels: (**a**) 1:0.25PEG(508); (**b**) 1:0.5PEG(508); (**c**) 1:0.25PEG(6000); and (**d**) 1:0.25PEG(20,000) (scale bar = 10 µm).

We investigated the internal structures of the p(NIPAm*-co-*PEGDA) hydrogels with different compositions by SEM. Several studies that accurately measured internal architectures of swollen hydrogels using cyro-SEM have been reported [26,27]. However, because relative differences by the composition of NIPAm and PEGDA were important, we used field emission SEM with a convienence. First, we varied the molecular weight of PEGDA to observe its effect on the internal microstructures of the hydrogels. As seen in Figure 3a,c,d, at a constant weight ratio of NIPAm to PEGDA, the pore size of the hydrogels increased with increasing molecular weight of PEGDA. On the other hand, at the same molecular weight of PEGDA, the pore size was reduced when the weight ratio was increased from 1:0.25 to 1:0.5 (Figure 3b). These SEM images suggest that the internal microstructures of the p(NIPAm*-co-*PEGDA) hydrogels could be controlled by manipulating the molecular weight of PEGDA or the weight ratios of NIPAm and PEGDA. Interestingly, some of the hydrogels, 1:0.25PEG(508) and 1:0.5PEG(508), seemed to have interconnected pores, and the pore sizes were within a few micrometers, while other hydrogels, such as 1:0.25PEG(6000) and 1:0.25PEG(20,000), exhibited larger pore sizes but closed pore structures. These observations indicated that an increase in the molecular weight of PEGDA decreased the molar percentage of PEGDA, and, as a result, decreased the crosslinking density of the hydrogels, which in turn increased the swelling capability of the hydrogels.

### 3.3. Effect of Molecular Weight of PEGDA on the Swelling Ratio

To investigate the effect of the molecular weight of PEGDA on the swelling properties of the p(NIPAm*-co-*PEGDA) hydrogels, we varied the molecular weight of PEGDA, while keeping the weight ratio of NIPAm to PEGDA constant. Previously, Padmavathi and Chatterji demonstrated that the length of a polymer chain between crosslinks (M_c_: molecular weight between crosslinks) decreases with increasing PEGDA concentration and vice versa [28]. Moreover, Weber et al. synthesized poly (ethylene glycol) dimethacrylate (PEGDM) hydrogels and showed that crosslinkable double bonds in PEGDM hydrogels decreased with increasing molecular weight of PEGDM, which in turn enhanced the swelling capability of the hydrogels [29].

**Figure 4:**
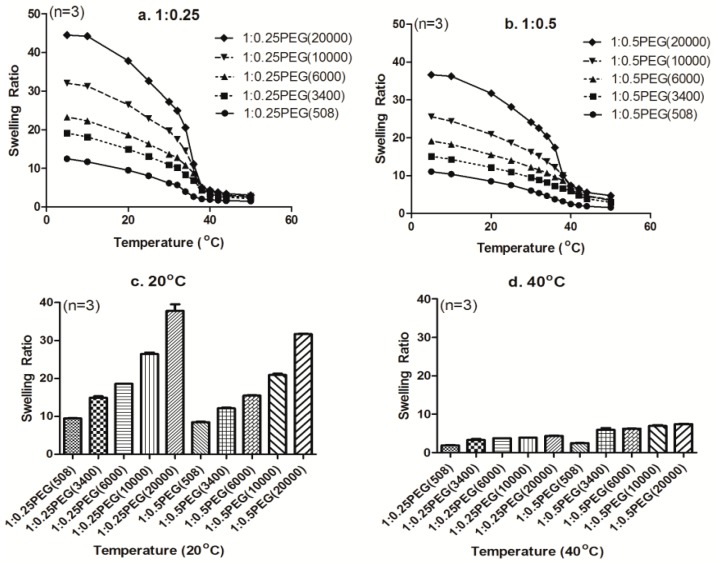
Swelling ratios of the p(NIPAm*-co-*PEGDA) hydrogels: (**a**) with weight ratio of NIPAm and PEGDA as 1:0.25; (**b**) with weight ratio of NIPAm and PEGDA as 1:0.5; (**c**,**d**) swelling ratios at two different temperatures (*n* = 3).

In our study, instead of the conventionally synthesized PNIPAm with *N*,*N′*-methylene bisacrylamide (MBAm) as a crosslinker, we introduced PEGDA onto NIPAm hydrogels to take advantage of its dual role as a crosslinker to tailor the crosslinking density of the hydrogels, as well as to provide hydrophilic moieties to the NIPAm network to tune their swelling behaviors. As seen in Figure 4, higher swelling ratios were observed for both weight ratios of p(NIPAm*-co-*PEGDA) hydrogels when the molecular weight of PEGDA was increased while maintaining a constant concentration. These observations clearly suggested that increasing the molecular weight of PEGDA decreased the molar percentage of PEGDA, and, as a result, decreased the crosslinking density of the hydrogels. At the same time, increasing the molecular weight of PEGDA would increase M_c_ and thereby increase the swelling capacity of the hydrogels. Furthermore, those results showed that a LCST of PEGs was modulated by their molecular weight and affected the LCST change of p(NIPAm*-co-*PEGDA) hydrogels. In fact, reports that the LCST is affected by the size of the side groups and varies depending on the length of the ethylene oxide chain support our conclusion [30,31,32,33,34,35].

### 3.4. Effect of PEGDA Concentration on the Swelling Ratio

**Figure 5:**
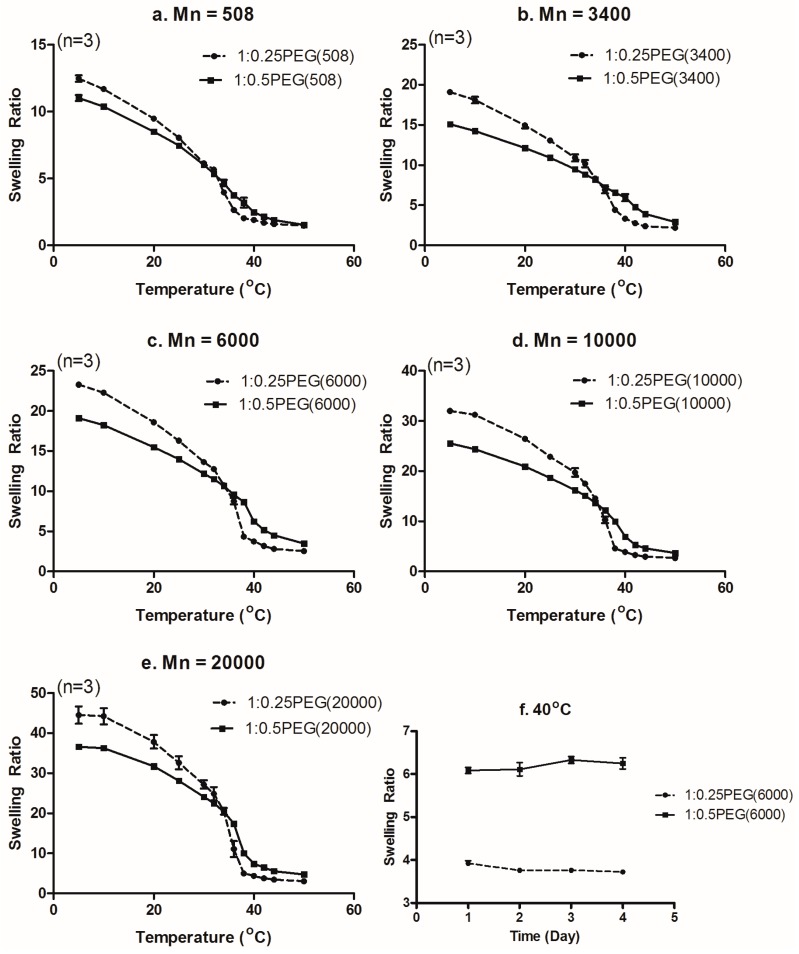
Comparison of the swelling ratio of the different p(NIPAm*-co-*PEGDA) hydrogels: (**a**–**e**) Between two different weight ratios with the same molecular weight of PEGDA; (**f**) Between 1:0.25PEG(6000) and 1:0.5PEG(6000) at 40 °C.

With the understanding of the effect of the molecular weight of PEGDA on the swelling behaviors of the p(NIPAm-*co*-PEGDA) hydrogels, we next investigated the effect of PEGDA concentration on swelling behaviors. Previously, Bryant et al. had characterized their PEGDM hydrogels and showed that increasing the crosslinking density of PEGDM hydrogels decreased their swelling capacity due to the decreased mesh sizes [36,37]. And Truong et al. showed that the equilibrium water content of PMAC(poly(5-Methyl-5-allyloxycarbonyl-1,3-dioxan-2-one))-PEG was decreased by a rise in temperature [26]. The reason for this is that swelling ratios of PEGs are decreased by dehydration as the temperature is increased. Actually, as shown in Figure 3 and Figure 4, swelling ratios of all p(NIPAm-*co*-PEGDA) hydrogels decreased at elevated temperature rise. In addition, when the weight ratio of NIPAm and PEGDA was increased from 1:0.25 to 1:0.5, while maintaining the same molecular weight of PEGDA, the swelling ratios of the p(NIPAm-*co*-PEGDA) hydrogels decreased in the lower temperature region and increased at higher temperatures (above the crossover points in Figure 5). By comparing the swelling ratios at two distinct temperatures, which were chosen because they were thought to be below and above the LCST, we evaluated the effect of PEGDA concentration on the swelling ratio of the hydrogels. 

At 20 °C, the swelling ratios of the hydrogels with higher amounts of PEGDA (1:0.5) were lower than those of their counterparts (1:0.25) (Figure 4c). These results indicated that increasing the amount of PEGDA leads to an increase in the crosslinking density of hydrogels, and, as a result, limits their swelling capacity. Interestingly, at 40 °C or above the crossover points, the swelling ratios of the hydrogels increased with increasing the amount of PEGDA at all molecular weights of PEGDA (Figure 4d and Figure 5a–e). To determine whether this phenomenon was due to diffusion rate-related deswelling kinetics, we compared the long-term swelling ratios of two hydrogels (1:0.25PEG(6000) and 1:0.5PEG(6000)). We equilibrated each hydrogel at 40 °C first and then measured their swollen weights over the next four consecutive days. As shown in Figure 5f, their swelling ratios did not change over four days of swelling in DI water. Our long-term observation at the equilibrium state showed that the diffusion rate-related deswelling kinetics was not related to the reversed swelling ratios over the crossover points. We therefore concluded that the reversed swelling ratios above a certain temperature (crossover points) would be mainly due to the increase in the number of hydrophilic oxyethylene side chains of PEGDA in the polymer network, which allows more water molecules to be bound to the hydrogel network and leads to increased swelling ratios. 

### 3.5. Kinetics of Deswelling/Reswelling

Previously, Okano and co-workers demonstrated that a skin layer is formed when PNIPAm hydrogels undergo deswelling above their LCST and that this prevents water molecules from coming out of the polymer network, which is why conventional PNIPAm hydrogels show slow deswelling kinetics [11,38]. 

**Figure 6:**
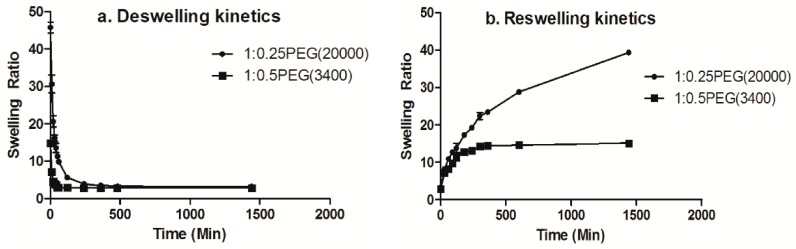
(**a**) Deswelling kinetics curve and (**b**) Reswelling kinetics curve of the 1:0.25PEG(20,000) and 1:0.5PEG(3400) hydrogels.

As seen in Figure 6, the 1:0.25PEG(6000) hydrogel in our study underwent an 80% decrease in its swelling ratio after immersion at 50 °C for hour, showing faster deswelling kinetics than the previously reported PNIPAm hydrogels, and the reswelling kinetics was also rapid [4]. These results suggested that the formation of a hydrophobic skin layer was hindered by the incorporation of a hydrophilic co-monomer PEGDA, which increased the deswelling kinetics, a result that is similar to that of previously reported studies [11].

### 3.6. Combined Effect of the Molecular Weight and Concentration of PEGDA on LCST

The LCST of each hydrogel was determined from the plot of the swelling ratios at different temperatures (Figure 4a,b), and the LCSTs are summarized in Table 2. With increasing weight ratios of PEGDA to NIPAm, the LCSTs of the hydrogels increased at the corresponding molecular weight of PEGDA. Except for the 1:0.25PEG(508) and 1:0.5PEG(508) hydrogels that were synthesized from liquid PEGDA (Mn = 508), the LCSTs at Mn = 3400, 6000, 10,000 and 20,000 with the same co-monomer ratio decreased with an increase in the molecular weight as a consequence of decreased number of hydrophilic oxyethylene groups [39]. Furthermore, at the same weight ratio of PEGDA, increasing the molecular weight of PEGDA decreased a molar percentage of PEGDA, and, as a result, decreased the crosslinking density of the hydrogels. Also, a decrease of the density increases spaces for a water uptake at the hydrogel. In addition, a low density of p(NIPAm-*co*-PEGDA) contributed to the decrease of the hydrophobic region within the gel structure and increased of the swelling capacity of the hydrogels. 

In our study, the thermo-sensitive behaviors of the polymer networks, such as the LCST, were found to be affected by several factors such as hydrophilic-hydrophobic balance, crosslinking density, and molecular weight of PEGDA. With a fixed weight fraction of PEGDA, the hydrophilicity of the polymer networks would be decreased, which may have resulted in a decrease in the LCST with increasing molecular weight of PEGDA. However, the swelling behaviors of the 1:0.25PEG(508) and 1:0.5PEG(508) hydrogels did not follow the same trend, which may have been caused by the phase difference in the PEGDA used in the 1:0.25PEG(508) and 1:0.5PEG(508) hydrogels. Additional studies will be required to determine the exact reason for this result.

### 3.7. Application of the Prepared Hydrogels as 2D Monolayer Cell Culture Substrates for Cell Sheet Engineering

**Figure 7:**
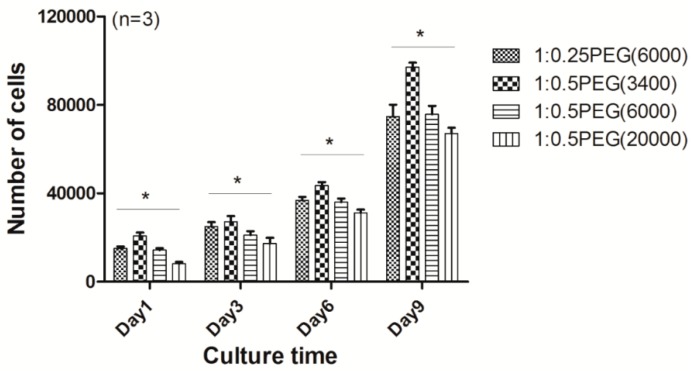
Proliferation of the seeded cells during nine days of in vitro culture (* *p* < 0.05).

To investigate the potential of the p(NIPAm*-co-*PEGDA) hydrogels for use as 2D monolayer cell culture substrates to detach confluent cells as intact cell sheet without using enzymes, we seeded bCCs on the gel surface. We then evaluated whether the hydrogels could support cell proliferation and whether there was difference in the cellular activities depending upon the compositions of the different hydrogels. The proliferation of the seeded cells on the hydrogel surface was examined by counting the number of cells as a function of culture time. As seen in Figure 7, the bCCs proliferated on all the hydrogels and significant differences were observed in the initial cell attachment, noticeably between the 1:0.5PEG(20,000) hydrogel and the 1:0.5PEG(3400) hydrogel. In addition to the different degrees of initial cell attachment, we observed that the 1:0.5PEG(3400) hydrogel provided the highest proliferation rate over the whole culture period and that cell proliferation decreased with increasing molecular weight at a NIPAm to PEGDA weight ratio of 1:0.5. These findings suggested that initial cell attachment and proliferation of the seeded cells were determined by the degree of hydrophilic-hydrophobic balance of the hydrogel surface. 

**Figure 8:**
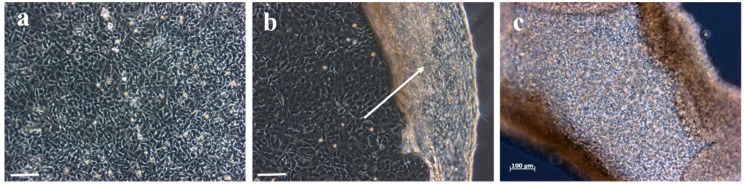
Phase contrast microscopic images of chondrocytes plated on the 1:0.5PEG(3400) hydrogel sheet showing: (**a**) confluent chondrocytes (**b**) partially detached chondrocytes as a cell sheet after exposure to room temperature (an arrow indicates the site of detachment) (**c**) completely detached chondrocytes as a cell sheet (scale bar = 50, 50, and 100 µm, respectively).

Microscopic images of confluent chondrocytes plated on the 1:0.5PEG(3400) hydrogel are shown in Figure 8a. After the cells became confluent, we exposed them to room temperature to see whether the intact cell sheet can be detached spontaneously upon changing the surrounding temperature. After around seven to ten minutes at room temperature, confluent chondrocytes started detaching from the edges and completely detached from the 1:0.5PEG(3400) hydrogel within a few minutes (Figure 8b,c). We therefore concluded that by changing the surrounding temperature, thermo-responsive p(NIPAm-*co*-PEGDA) hydrogels used as monolayer cell culture substrates can be made to spontaneously detach confluent cells as intact cell sheets due to changes in the hydrophilic-hydrophobic balance of the hydrogel surfaces. These results confirmed our hypothesis that our hydrogels can be utilized as cell sheets with enhanced cell proliferation performance.

## 4. Conclusions

We synthesized thermo-responsive p(NIPAm*-co-*PEGDA) hydrogels by introducing PEGDA as a hydrophilic co-monomer as well as a crosslinker. By changing the molecular weight of PEGDA and the weight ratio of NIPAm to PEGDA, we could manipulate the swelling ratio of the hydrogels. By varying the molecular weight and amount of PEGDA in p(NIPAm*-co-*PEGDA), we could manipulate the length of the polymer chain between the crosslinks, the crosslink density, and the hydrophilic-hydrophobic balance of hydrogels, which is reflected in the swelling properties of the resultant hydrogels. In addition, we demonstrated the potential of the p(NIPAm*-co-*PEGDA) hydrogels for use as 2D monolayer cell culture substrates by culturing bovine chondrocytes on the hydrogels. Our hydrogels could support cell proliferation over the entire culture period, and confluent chondrocytes were spontaneously detached upon decreasing the surrounding temperature to room temperature.

## Figures and Tables

**Table 1:** Compositions and nomenclatures of the synthesized poly(*N*-isopropylacrylamide) with poly(ethylene glycol) diacrylate (p(NIPAm*-co-*PEGDA)) hydrogels.

Sample Codes	Molecular Weights of PEGDA	Weight Ratios (NIPAm:PEGDA)	Molar Ratios (NIPAm:PEGDA)
1:0.25PEG(508)/1:0.5PEG(508)	508	1:0.25/1:0.5	1:0.0557/1:0.1114
1:0.25PEG(3400)/1:0.5PEG(3400)	3400	1:0.25/1:0.5	1:0.0083/1:0.0166
1:0.25PEG(6000)/1:0.5PEG(6000)	6000	1:0.25/1:0.5	1:0.0047/1:0.0094
1:0.25PEG(10,000)/1:0.5PEG(10,000)	10,000	1:0.25/1:0.5	1:0.0028/1:0.0057
1:0.25PEG(20,000)/1:0.5PEG(20,000)	20,000	1:0.25/1:0.5	1:0.0014/1:0.0028

**Table 2:** Lower critical solution temperatures (LCSTs) of the p(NIPAm*-co-*PEGDA) hydrogels.

NIPAM:PEGDA Weight Ratio	Molecular Weight of PEGDA				
	508	3400	6000	10,000	20,000
1:0.25	34.0 ± 0.3 °C	36.3 ± 0.3 °C	36.3 ± 0.3 °C	35.9 ± 0.1 °C	35.3 ± 0.2 °C
1:0.5	34.3 ±0.3 °C	41.4 ± 0.3 °C	39.1 ± 0.3 °C	38.5 ± 0.5 °C	36.9 ± 0.4 °C
